# Changes in lipoprotein lipase and endothelial lipase mass in familial hypercholesterolemia during three-drug lipid-lowering combination therapy

**DOI:** 10.1186/s12944-016-0238-z

**Published:** 2016-04-02

**Authors:** Hayato Tada, Junji Kobayashi, Masa-aki Kawashiri, Kazuya Miyashita, Atsushi Nohara, Akihiro Inazu, Katsuyuki Nakajima, Hiroshi Mabuchi, Masakazu Yamagishi

**Affiliations:** Division of Cardiovascular Medicine, Kanazawa University Graduate School of Medicine, 13-1 Takara-machi, Kanazawa, 920-8641 Japan; Department of General Medicine, Kanazawa Medical University, Uchinada, Japan; Immuno-Biological Laboratories Co., Ltd, Fujioka, Japan; Department of Advanced Research in Community Medicine, Kanazawa University Graduate School of Medical Science, Kanazawa, Japan; Department of Laboratory Science, Molecular Biochemistry and Molecular Biology, Graduate School of Medical Science, Kanazawa University, Kanazawa, Japan; Department of Laboratory Sciences, Gunma University Graduate School of Health Sciences, Maebashi, Japan

**Keywords:** Lipoprotein lipase, Endothelial lipase, Familial hypercholesterolemia

## Abstract

**Background:**

This study was performed to compare the effects of three different lipid-lowering therapies (statins, ezetimibe, and colestimide) on lipoprotein lipase and endothelial lipase masses in pre-heparin plasma (pre-heparin *LPL* and *EL* mass, respectively) from patients with familial hypercholesterolemia (FH). FH is usually treated by coadministration of these three drugs.

**Methods:**

The pre-heparin *LPL* and *EL* masses were measured in fresh frozen plasma drawn and stored at various time points during coadministration of the three drugs from patients with heterozygous FH harboring a single mutation in the LDL receptor (*n* = 16, mean age 63 years). The patients were randomly divided into two groups based on the timing when ezetimibe was added.

**Results:**

Plasma *LPL* mass concentration was significantly reduced by rosuvastatin at 20 mg/day (median = 87.4 [IQR: 71.4–124.7] to 67.5 [IQR: 62.1–114.3] ng/ml, *P* < 0.05). In contrast, ezetimibe at 10 mg/day as well as colestimide at 3.62 g/day did not alter its level substantially (median = 67.5 [IQR: 62.1–114.3] to 70.2 [IQR: 58.3–106.2], and to 74.9 [IQR: 55.6–101.3] ng/ml, respectively) in the group starting with rosuvastatin followed by the addition of ezetimibe and colestimide. On the other hand, the magnitude in *LPL* mass reduction was lower in the group starting with ezetimibe at 10 mg/day before reaching the maximum dose of 20 mg/day of rosuvastatin. Plasma *EL* mass concentration was significantly increased by rosuvastatin at 20 mg/day (median = 278.8 [IQR: 186.7–288.7] to 297.0 [IQR: 266.2–300.2] ng/ml, *P* < 0.05), whereas other drugs did not significantly alter its level.

**Conclusion:**

The effects on changes of *LPL* and *EL* mass differed depending on the lipid-lowering therapy, which may impact the prevention of atherosclerosis differently.

**Electronic supplementary material:**

The online version of this article (doi:10.1186/s12944-016-0238-z) contains supplementary material, which is available to authorized users.

## Background

Lipoprotein lipase (*LPL*) and endothelial lipase (*EL*) hydrolyze triglyceride (TG) in circulating chylomicrons and very low-density lipoprotein (VLDL) on the surface of endothelial cells [1]. It has been demonstrated that a reduced concentration of plasma *LPL* mass is associated with an increased risk of coronary artery disease [2, 3]. Several studies have demonstrated that plasma *LPL* mass concentration could be altered by drug manipulations, such as fibrate, insulin sensitizer, and statins in patients with diabetes, potentially affecting the progression of their atherosclerosis [4–6]. In addition, recent genetic studies have suggested that genetic modulators of *LPL* mediate cardiovascular risk. For example, loss-of-function mutations in apolipoprotein C3 (*APOC3*), which is an inhibitor of *LPL*, decrease the risk of coronary artery disease (CAD) [7], while loss-of-function mutations in apolipoprotein A5 (*APOA5*), which is an activator of *LPL*, increases the risk of CAD [8]. In contrast, large-scale clinical trials assessing the efficacy of HDL cholesterol-raising therapies have failed [9, 10] based on the neutral impact of HDL cholesterol concentration on CAD estimated by Mendelian randomization studies [11, 12]. Accordingly, targeting plasma *LPL* (and *EL*) concentrations, rather than plasma HDL cholesterol concentration, could be much more reasonable in terms of preventive cardiology. However, no study has yet assessed the changes in plasma lipase mass concentration during lipid-lowering therapy in patients with familial hypercholesterolemia (FH), which usually requires the coadministration of different types of such drugs [13]. Therefore, we compared the effects of three different lipid-lowering therapies (statins, ezetimibe, and colestimide) on *LPL* and *EL* mass in pre-heparin plasma (pre-heparin *LPL* and *EL* mass, respectively) in patients with FH.

## Methods

### Study subjects

**Fig. 1:**
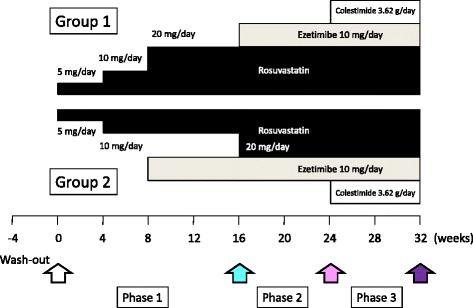
Study design. The present study was conducted as a prospective open randomized study to investigate the efficacy and safety of coadministration of rosuvastatin at 20 mg/day, ezetimibe at 10 mg/day, and granulated colestimide at 3.62 g/day. Any lipid-lowering agents had been washed out ≥ 4 weeks before entry into the study. Study subjects were divided into two groups by an envelope-based method to elucidate the secondary end point of the present study: rosuvastatin at 20 mg/day (group 1) versus rosuvastatin at 10 mg/day coadministered with ezetimibe at 10 mg/day (group 2). The *white*, *blue*, *pink*, and *purple arrows* indicate baseline, second, third, and last blood sampling, respectively

The study population consisted of patients with heterozygous FH who participated in a previous clinical trial conducted as a prospective open randomized study to investigate the efficacy and safety of coadministration of rosuvastatin (20 mg/day), ezetimibe (10 mg/day), and granulated colestimide (3.62 g/day) at the maximum doses permitted in Japan. All 17 subjects were heterozygous with a confirmed LDL receptor gene mutation and fulfilled our clinical diagnostic criteria for heterozygous FH: patients with primary hyper-LDL cholesterolemia (>160 mg/dl) with tendon xanthoma or those with first-degree relatives with previously diagnosed heterozygous FH showing primary hyper-LDL cholesterolemia (>160 mg/dl). Exclusion criteria of the present study were FH patients with a homozygous gene mutation, patients under LDL apheresis therapy or any immunomodulatory medication, patients with fasting serum triglyceride levels >500 mg/dl, patients with hepatic disease, or patients within 12 weeks after the onset of an acute myocardial infarction or stroke. Details of this study have been described elsewhere [14], and 16 of 17 patients whose fresh frozen plasma was available were included in this study. The subjects were divided into two groups using a sealed envelope-based method according to the timing when ezetimibe was added at 10 mg/day. All participants were started on a 4-week treatment with rosuvastatin at 5 mg/day followed by another 4-week treatment of rosuvastatin at 10 mg/day. The dose of rosuvastatin in group 1 was increased to 20 mg with an 8-week follow-up, whereas group 2 received ezetimibe at 10 mg/day with an 8-week follow-up (phase 1). After phase 1, group 1 received ezetimibe at 10 mg/day added to rosuvastatin for 8 weeks, whereas in group 2 the doses of rosuvastatin were increased to 20 mg with an 8-week follow-up (phase 2). In phase 3, groups 1 and 2 were given 3.62 g of granulated colestimide (twice a day before meals, once in the morning and once in the evening) added to the phase 2 treatment regimen (Fig. 1).

### Determination of *LPL* and *EL* mass concentrations

Fasting blood samples were drawn and stored at −80 °C during the study period. Pre-heparin *LPL* concentrations were determined with a highly sensitive and specific ELISA kit (Code No.27184; IBL, Fujioka, Japan) [15, 16]. *EL* mass concentrations were determined using our ELISA system (Code No. 27182; IBL) [17].

### Biochemical analysis

Fasting blood samples were drawn for assays. Plasma concentrations of total cholesterol, TG, and HDL cholesterol were determined enzymatically as described previously [18]. Remnant-like particles (RLP) were determined as described previously [19].

### Statistical analysis

Categorical variables are expressed as percentages. Continuous variables with a normal distribution are shown as means (± SD), and those with a skewed distribution are shown as medians (interquartile [IQR]). The changes in each lipase mass concentration were compared by analysis of variance (ANOVA). All statistical analyses were conducted using R. In all analyses, *P* < 0.05 was assumed to indicate statistical significance.

### Ethical considerations

This study was approved by the Ethics Committees of Kanazawa University (Number: 1883–1) and Kanazawa Medical University (Number: 272). All procedures were performed in accordance with the ethical standards of the responsible committee on human experimentation (institutional and national) and with the Helsinki Declaration of 1975, as revised in 2008. Informed consent for genetic analyses was obtained from subjects with FH for inclusion in the study.

## Results

### Baseline characteristics of study subjects

Sixteen Japanese subjects with heterozygous FH were enrolled in the present study. Baseline characteristics and concomitant drug therapies are listed in Table 1. Five of six diabetic patients (28 %) that were under hypoglycemic medical therapy had a glycohemoglobin concentration < 7.0 %. No patients were treated with insulin injection therapy. Dosages of coadministered medications were kept constant during the entire study period.

**Table 1:** Baseline characteristics

	All (*n* = 16)	Group 1 (*n* = 9)	Group 2 (*n* = 7)
Age (yr)	63 ± 10	60 ± 11	68 ± 7
Male (n,%)	11 (69 %)	4 (44 %)	5 (71 %)
Body mass index (kg/m^2^)	23.7 ± 2.0	23.1 ± 1.9	24.5 ± 1.9
Total cholesterol (mg/dl)	380 ± 42	394 ± 56	364 ± 51
Triglyceride (mg/dl)	95 [84–127]	95 [88–126]	91 [68–136]
HDL cholesterol (mg/dl)	46 ± 10	49 ± 14	43 ± 6
LDL cholesterol (mg/dl)	299 ± 48	309 ± 46	283 ± 48
RLP cholesterol (mg/dl)	8.9 ± 3.3	8.7 ± 3.5	9.2 ± 3.0
Hypertension (n, %)	8 (50 %)	3 (33 %)	5 (71 %)
Diabetes (n, %)	6 (38 %)	3 (33 %)	3 (43 %)
Smoking (n, %)	1 (6 %)	0 (0 %)	1 (14 %)
Coronary artery disease (n, %)	9 (56 %)	4 (44 %)	5 (71 %)

*RLP* remnant-like particle

### Changes in *LPL* mass during coadministration of three drugs

**Fig. 2:**
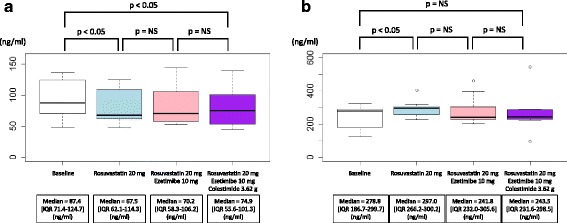
Lipase mass concentration during coadministration of three drugs in group 1. **a** *LPL* mass concentration. **b** *EL* mass concentration. White indicates the baseline. *Blue* indicates the second blood sampling when rosuvastatin was administered at 20 mg/day. *Pink* indicates the third blood sampling when rosuvastatin at 20 mg/day and ezetimibe at 10 mg/day were coadministered. *Purple* indicates the last blood sampling when rosuvastatin at 20 mg/day, ezetimibe at 10 mg/day, and colestimide at 3.62 g/day were coadministered

**Fig. 3:**
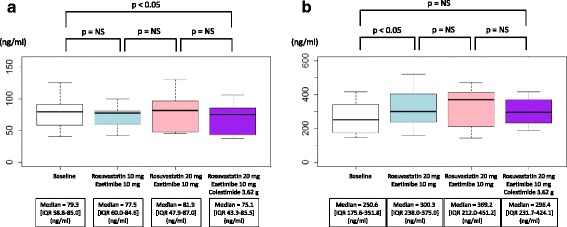
Lipase mass concentration during coadministration of three drugs in group 2. **a** *LPL* mass concentration. **b** *EL* mass concentration. White indicates the baseline. *Blue* indicates the second blood sampling when rosuvastatin at 10 mg/day and ezetimibe at 10 mg/day were coadministered. *Pink* indicates the third blood sampling when rosuvastatin at 20 mg/day and ezetimibe at 10 mg/day were coadministered. *Purple* indicates the last blood sampling when rosuvastatin at 20 mg/day, ezetimibe at 10 mg/day, and colestimide at 3.62 g/day were coadministered

In group 1, the *LPL* mass concentration was significantly reduced by rosuvastatin at 20 mg/day (median = 87.4 [IQR: 71.4–124.7] to 67.5 [IQR: 62.1–.3] ng/ml, *P* < 0.05, Fig. 2a), whereas ezetimibe at 10 mg/day as well as colestimide at 3.62 g/day did not markedly alter its level (median = 67.5 [IQR: 62.1–114.3] to 70.2 [IQR: 58.3–106.2], and to 74.9 [IQR: 55.6–101.3] ng/ml, respectively, *P* = NS, Fig. 2a). Similar trends were observed in group 2, in which the statistical significance of the reduction during phase 1 was diminished using combination therapy consisting of rosuvastatin at 10 mg/day and ezetimibe at 10 mg/day (median = 79.3 [IQR: 58.8–85.0] to 77.5 [IQR: 60.0–84.6] ng/ml, *P* = NS, Fig. 3a). On the other hand, the significant reduction in *LPL* mass concentration achieved by adding colestimide at 3.62 g/day was replicated in group 2 (median = 81.9 [IQR: 47.9–87.0] to 75.1 [IQR: 43.3–85.5] ng/ml, *P* < 0.05, Fig. 3a).

### Changes in *EL* mass during coadministration of three drugs

In group 1, the *EL* mass concentration was significantly increased by rosuvastatin at 20 mg/day (median = 278.8 [IQR: 186.7–288.7] to 297 [IQR: 266.2–300.2] ng/ml, *P* < 0.05, Fig. 2b), whereas ezetimibe at 10 mg/day as well as colestimide at 3.62 g/day did not markedly change its level (median = 297 [IQR: 266.2–300.2] to 241.8 [IQR: 232.0–305.6], and to 243.5 [IQR: 231.6–298.5] ng/ml, respectively, *P* = NS, Fig. 2b). In group 2, combination therapy with rosuvastatin at 10 mg/day and ezetimibe at 10 mg/day also increased its level significantly (median = 250.6 [IQR: 175.6–351.8] to 300.3 [IQR: 238.0–375.0] ng/ml, *P* < 0.05, Fig. 3b). On the other hand, there were no significant changes in EL mass concentration when ezetimibe was added at 10 mg/day and colestimide at 3.62 g/day (median = 300.3 [IQR: 238.0–375.0] to 369.2 [IQR: 212.0–451.2], and to 296.3 [IQR: 231.7–424.1] ng/ml, respectively, *P* = NS, Fig. 3b).

### Association between plasma lipids and lipase mass

We investigated the associations between changes in *LPL*/*EL* and those in LDL cholesterol, TG, or RLP cholesterol during each phase. There were no significant associations between the changes in *LPL* mass concentrations and those in lipids (Additional file 1: Figures S1–S6). Significant associations between the changes in *EL* mass concentrations and TG/RLP cholesterol were observed during phase 2 in group 2 (Additional file 1: Figures S7–S12).

## Discussion

We measured the pre-heparin *LPL* and *EL* mass at each time point during coadministration of three drugs (statin, ezetimibe, and colestimide) in patients with heterozygous FH harboring a single mutation in the LDL receptor to compare the effects of these drugs on the plasma lipase mass concentration. Our results indicate that (1) statin and colestimide significantly reduced plasma *LPL* mass, but ezetimibe did not alter its level; (2) statin significantly increased plasma *EL* mass, but ezetimibe and colestimide did not change its level; (3) there was no clear association between changes in lipase mass and changes in plasma lipid levels.

Recent Mendelian randomization trials have suggested that plasma TG is one of the causal factors for the development of coronary artery disease, rather than merely a marker [7, 20]. In addition, plasma TG level is one of the residual risks in this statin era [21]. Accordingly, reassessment of TG-rich lipoprotein metabolism seems to be a reasonable strategy to combat such residual risk. In this regard, genetic studies have indicated that the *LPL* and *APOC3* pathway is strongly associated with plasma TG as well as coronary artery disease, gathering a great deal of attention as a novel therapeutic target. Increasing plasma *LPL* concentration as well as reducing plasma *APOC3*, rather than increasing plasma HDL cholesterol, could be beneficial targets.

The results of one study that investigated the effect of statins on changes in *LPL* mass concentration in patients with diabetes showed results that were contrary to ours [6]. This difference may have been because we used the maximum dose of rosuvastatin in patients with FH whose TG-rich lipoprotein levels were shown to be impaired.

Our study has several limitations. First, the size of the study population was small because of the rarity of the disease (diagnosed genetic heterozygous FH). However, we observed similar tendencies between the two groups divided according to the timing of the initiation of ezetimibe. Second, this study did not have a crossover design, which could potentially lead to biased assessment of the differences among drugs. Third, we measured pre-heparin lipase mass levels instead of post-heparin levels, which could potentially affect the results, especially *LPL* mass levels.

## Conclusion

In conclusion, the effects on changes of *LPL* and *EL* mass were different depending on the lipid-lowering therapy, which may have different impacts on the preventive effects of the therapy on atherosclerosis.

## Additional file

Additional file 1: Associations between the changes in LPL/EL mass and those in lipids. (PPTX 2501 kb)

## Abbreviations

ANOVA: Analysis of variance
APOA5: Apolipoprotein A5
APOC3: Apolipoprotein C3
CAD: Coronary artery disease
EL: Endothelial lipase
FH: Familial hypercholesterolemia
IQR: Interquartile
LPL: Lipoprotein lipase
RLP: Remnant-like particle
TG: Triglyceride
VLDL: Very low-density lipoprotein
