# Supplementary material for: Changes in lipoprotein lipase and endothelial lipase mass in familial hypercholesterolemia during three-drug lipid-lowering combination therapy
Source: Lipids Health Dis. 2016 Apr 2;15:66. doi: 10.1186/s12944-016-0238-z (PMC4818918; doi:10.1186/s12944-016-0238-z)
Supplement: Additional file 1: — Associations between the changes in LPL/EL mass and those in lipids. (PPTX 2501 kb) [file 12944_2016_238_MOESM1_ESM.pptx]

## Slide 1
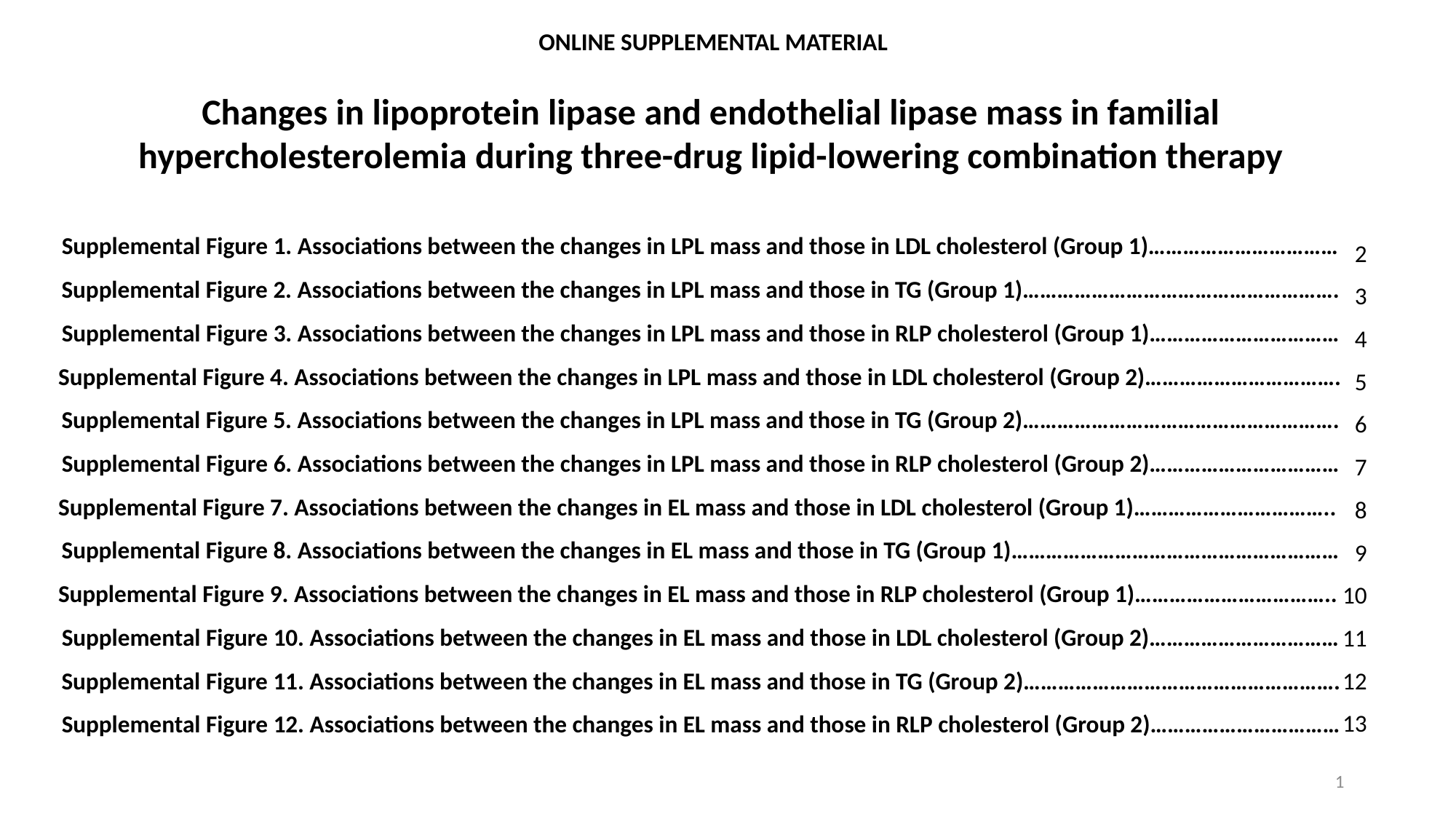

ONLINE SUPPLEMENTAL MATERIAL
Changes in lipoprotein lipase and endothelial lipase mass in familial hypercholesterolemia during three-drug lipid-lowering combination therapy
Supplemental Figure 1. Associations between the changes in LPL mass and those in LDL cholesterol (Group 1)……………………………
2
3
4
5
6
7
8
9
10
11
12
13
Supplemental Figure 2. Associations between the changes in LPL mass and those in TG (Group 1)……………………………………………….
Supplemental Figure 3. Associations between the changes in LPL mass and those in RLP cholesterol (Group 1)……………………………
Supplemental Figure 4. Associations between the changes in LPL mass and those in LDL cholesterol (Group 2)…………………………….
Supplemental Figure 5. Associations between the changes in LPL mass and those in TG (Group 2)……………………………………………….
Supplemental Figure 6. Associations between the changes in LPL mass and those in RLP cholesterol (Group 2)……………………………
Supplemental Figure 7. Associations between the changes in EL mass and those in LDL cholesterol (Group 1)……………………………..
Supplemental Figure 8. Associations between the changes in EL mass and those in TG (Group 1)…………………………………………………
Supplemental Figure 9. Associations between the changes in EL mass and those in RLP cholesterol (Group 1)……………………………..
Supplemental Figure 10. Associations between the changes in EL mass and those in LDL cholesterol (Group 2)……………………………
Supplemental Figure 11. Associations between the changes in EL mass and those in TG (Group 2)……………………………………………….
Supplemental Figure 12. Associations between the changes in EL mass and those in RLP cholesterol (Group 2)……………………………
1

## Slide 2
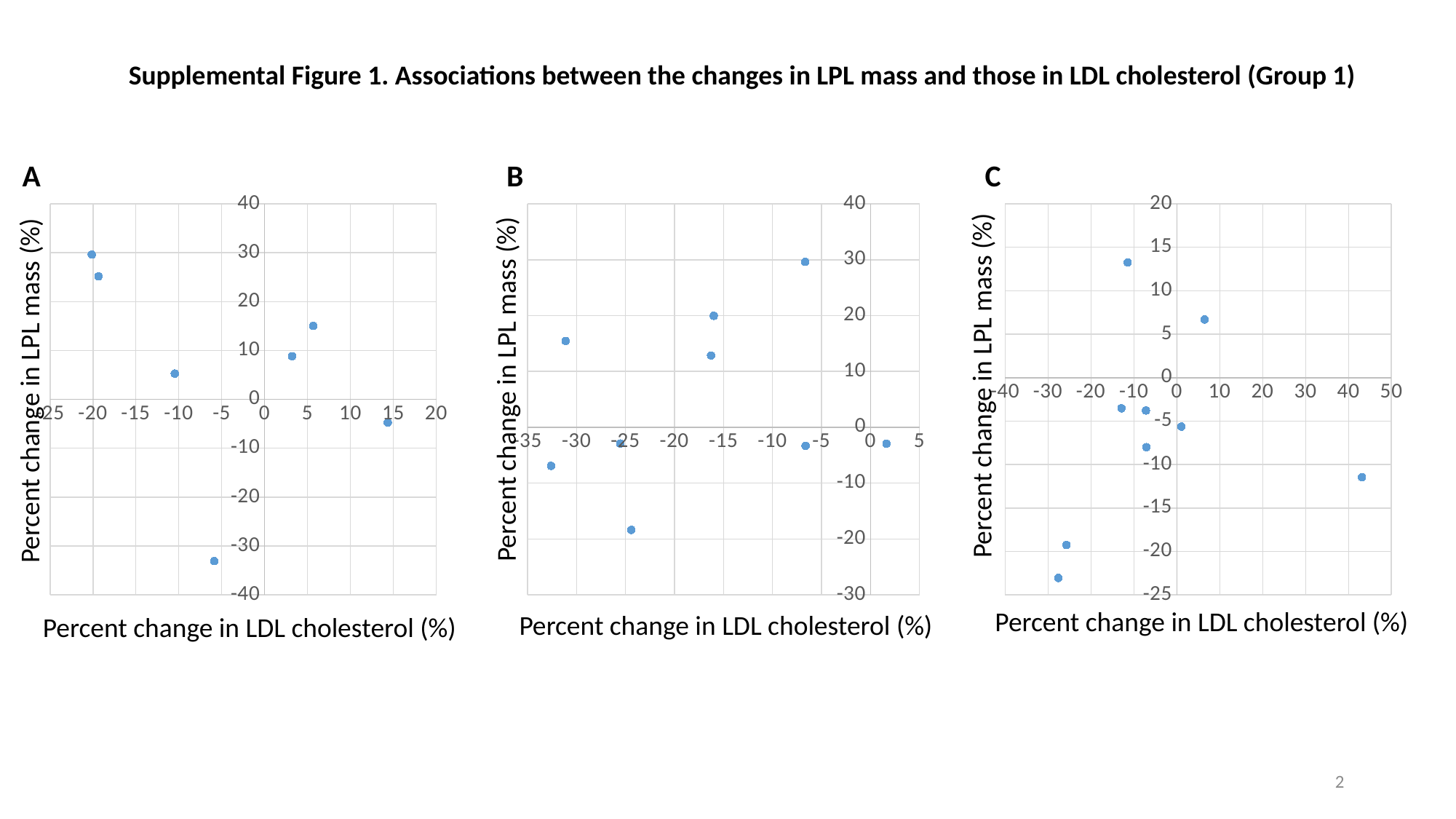

Supplemental Figure 1. Associations between the changes in LPL mass and those in LDL cholesterol (Group 1)
A
B
C
### Chart
| Category | |
|---|---|
### Chart
| Category | |
|---|---|
### Chart
| Category | |
|---|---|Percent change in LPL mass (%)
Percent change in LPL mass (%)
Percent change in LPL mass (%)
Percent change in LDL cholesterol (%)
Percent change in LDL cholesterol (%)
Percent change in LDL cholesterol (%)
2

## Slide 3
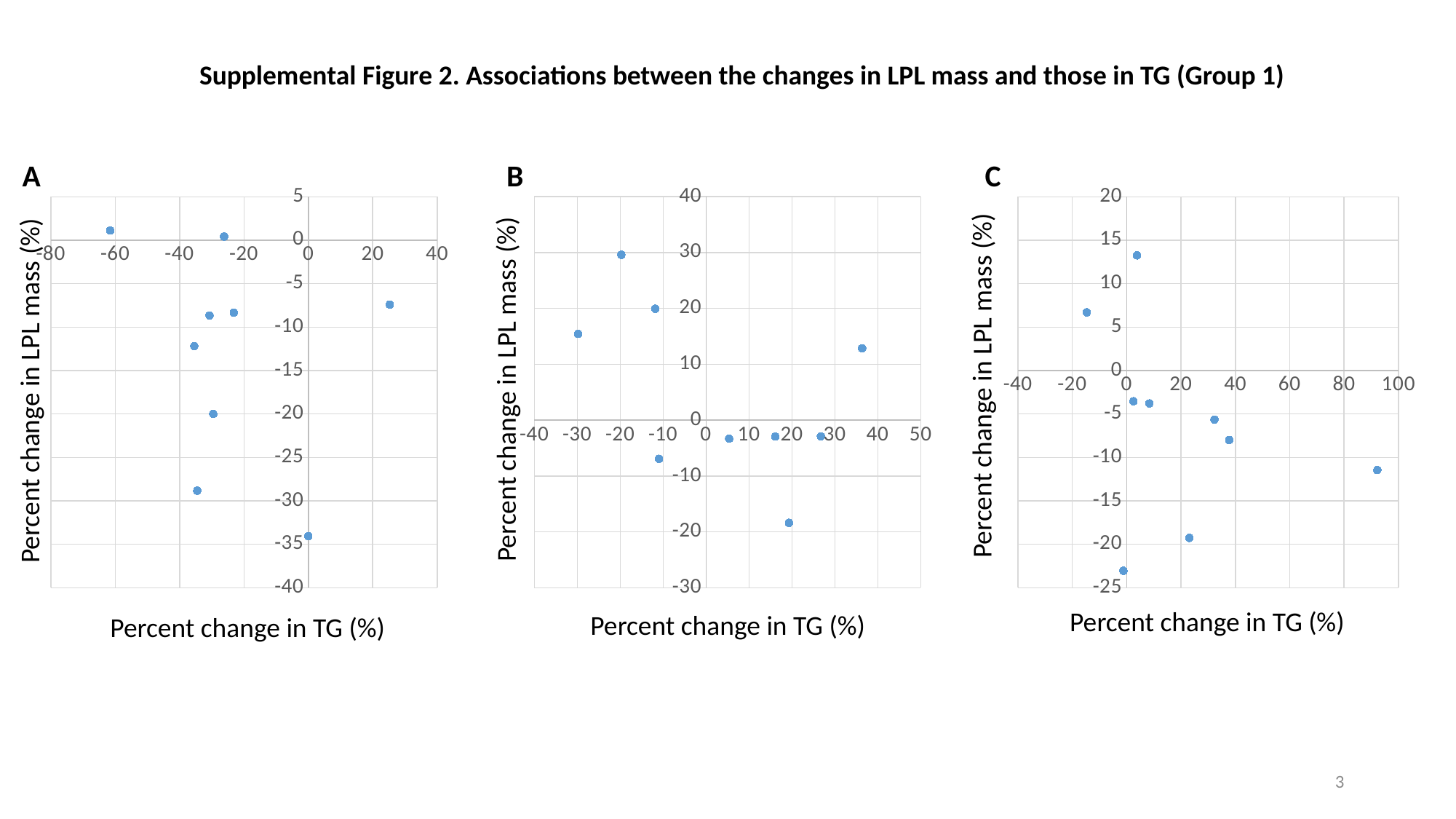

Supplemental Figure 2. Associations between the changes in LPL mass and those in TG (Group 1)
A
B
C
### Chart
| Category | |
|---|---|
### Chart
| Category | |
|---|---|
### Chart
| Category | |
|---|---|Percent change in LPL mass (%)
Percent change in LPL mass (%)
Percent change in LPL mass (%)
Percent change in TG (%)
Percent change in TG (%)
Percent change in TG (%)
3

## Slide 4
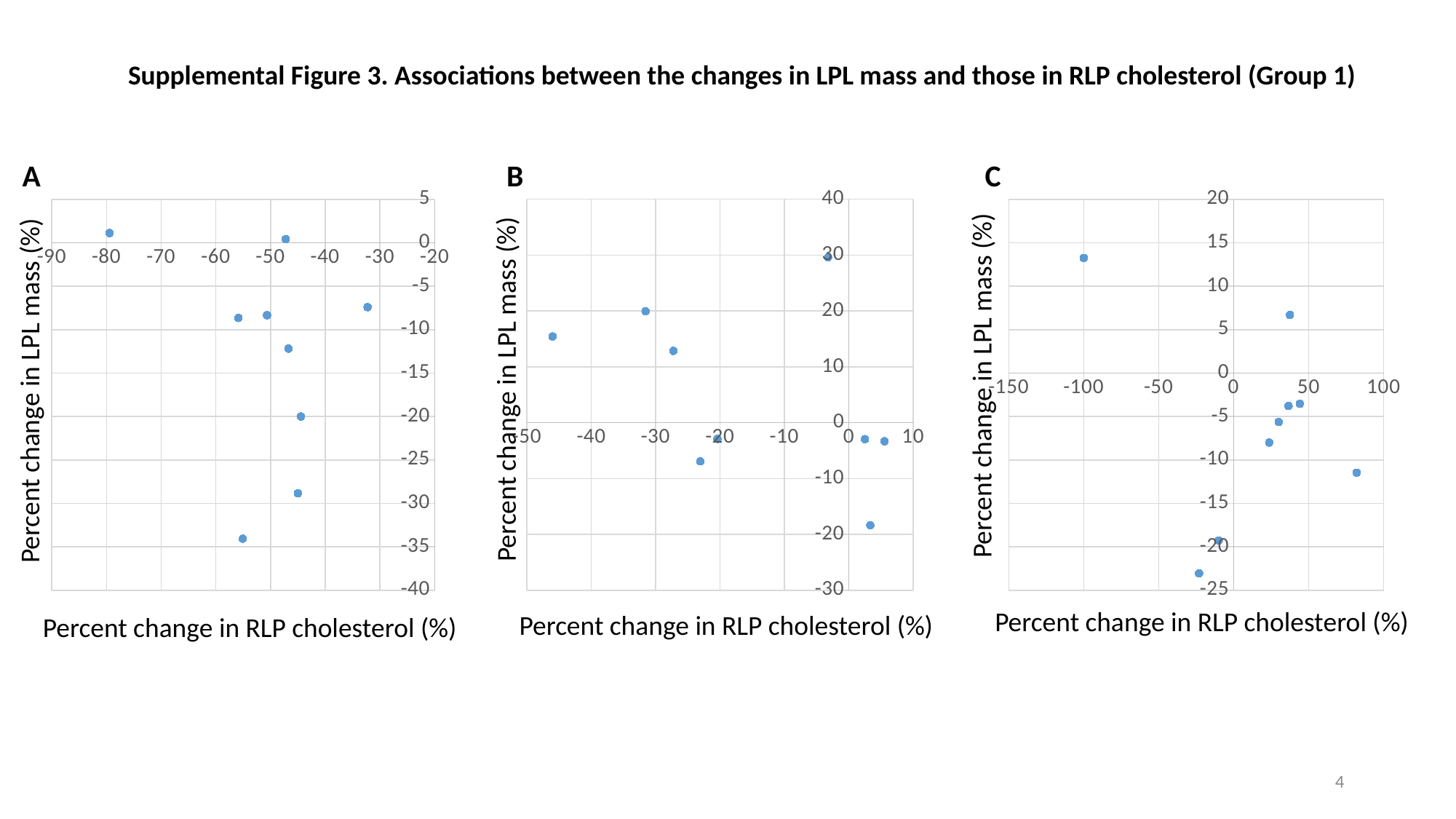

Supplemental Figure 3. Associations between the changes in LPL mass and those in RLP cholesterol (Group 1)
A
B
C
### Chart
| Category | |
|---|---|
### Chart
| Category | |
|---|---|
### Chart
| Category | |
|---|---|Percent change in LPL mass (%)
Percent change in LPL mass (%)
Percent change in LPL mass (%)
Percent change in RLP cholesterol (%)
Percent change in RLP cholesterol (%)
Percent change in RLP cholesterol (%)
4

## Slide 5
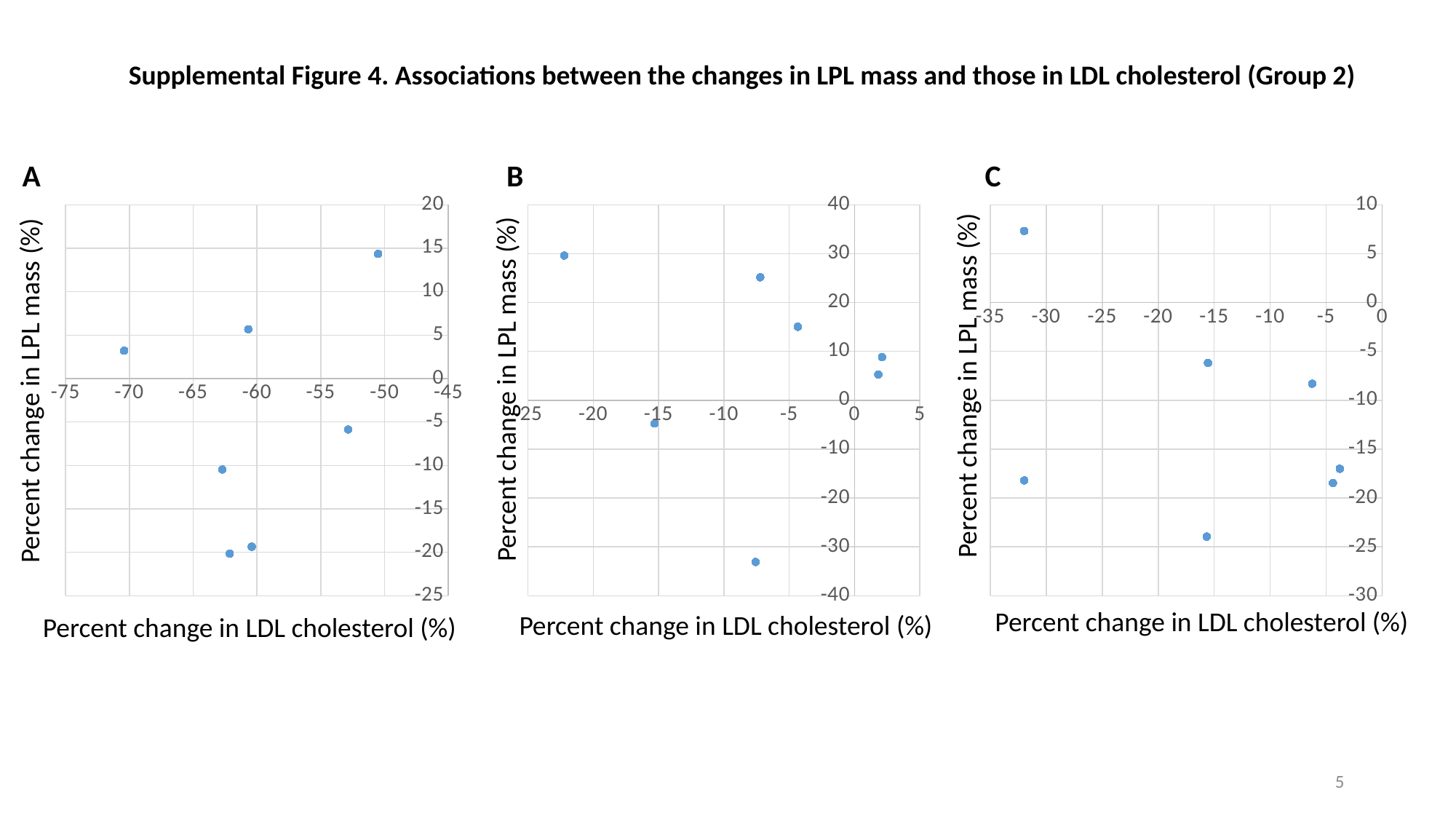

Supplemental Figure 4. Associations between the changes in LPL mass and those in LDL cholesterol (Group 2)
A
B
C
### Chart
| Category | |
|---|---|
### Chart
| Category | |
|---|---|
### Chart
| Category | |
|---|---|Percent change in LPL mass (%)
Percent change in LPL mass (%)
Percent change in LPL mass (%)
Percent change in LDL cholesterol (%)
Percent change in LDL cholesterol (%)
Percent change in LDL cholesterol (%)
5

## Slide 6
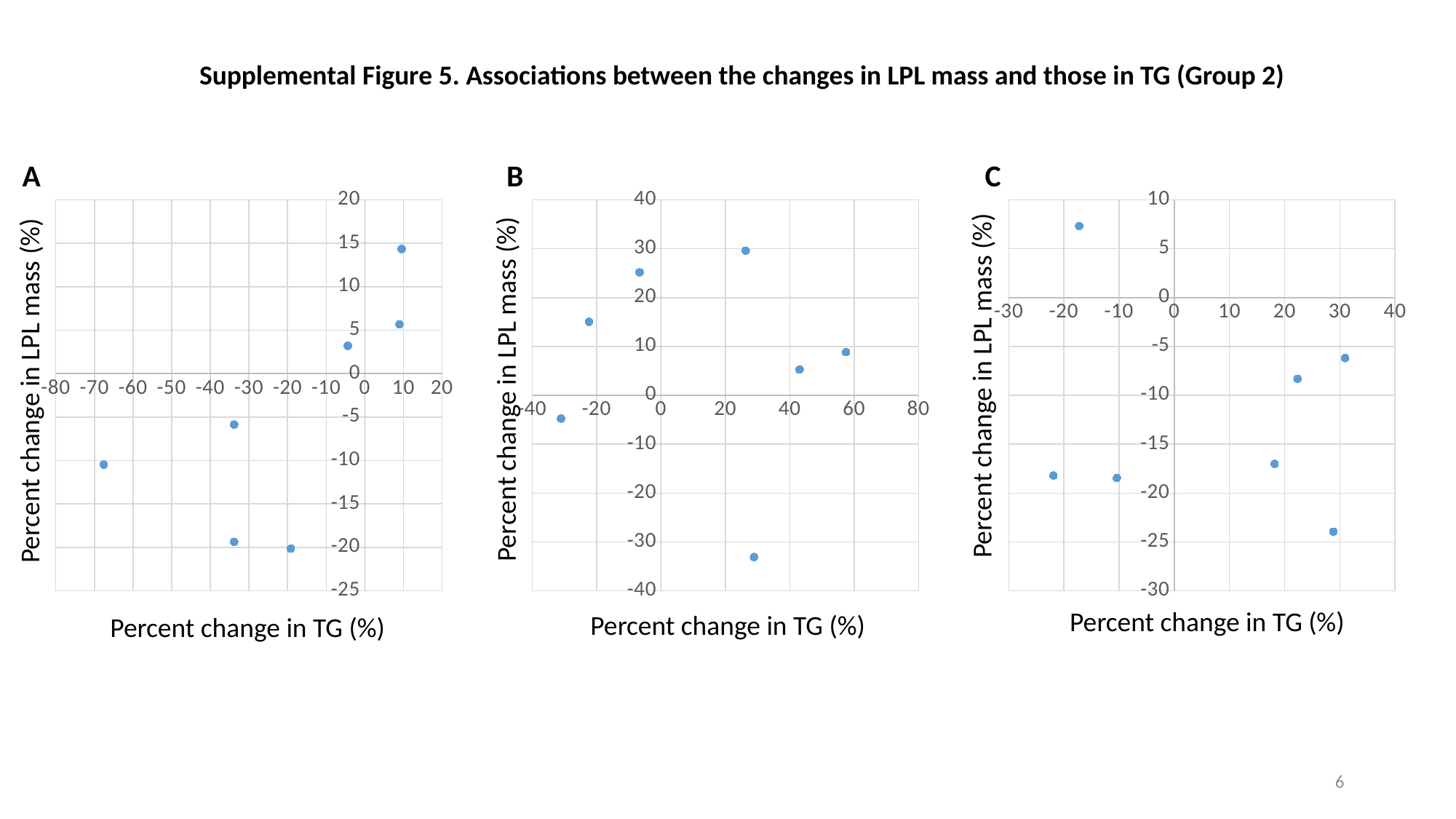

Supplemental Figure 5. Associations between the changes in LPL mass and those in TG (Group 2)
A
B
C
### Chart
| Category | |
|---|---|
### Chart
| Category | |
|---|---|
### Chart
| Category | |
|---|---|Percent change in LPL mass (%)
Percent change in LPL mass (%)
Percent change in LPL mass (%)
Percent change in TG (%)
Percent change in TG (%)
Percent change in TG (%)
6

## Slide 7
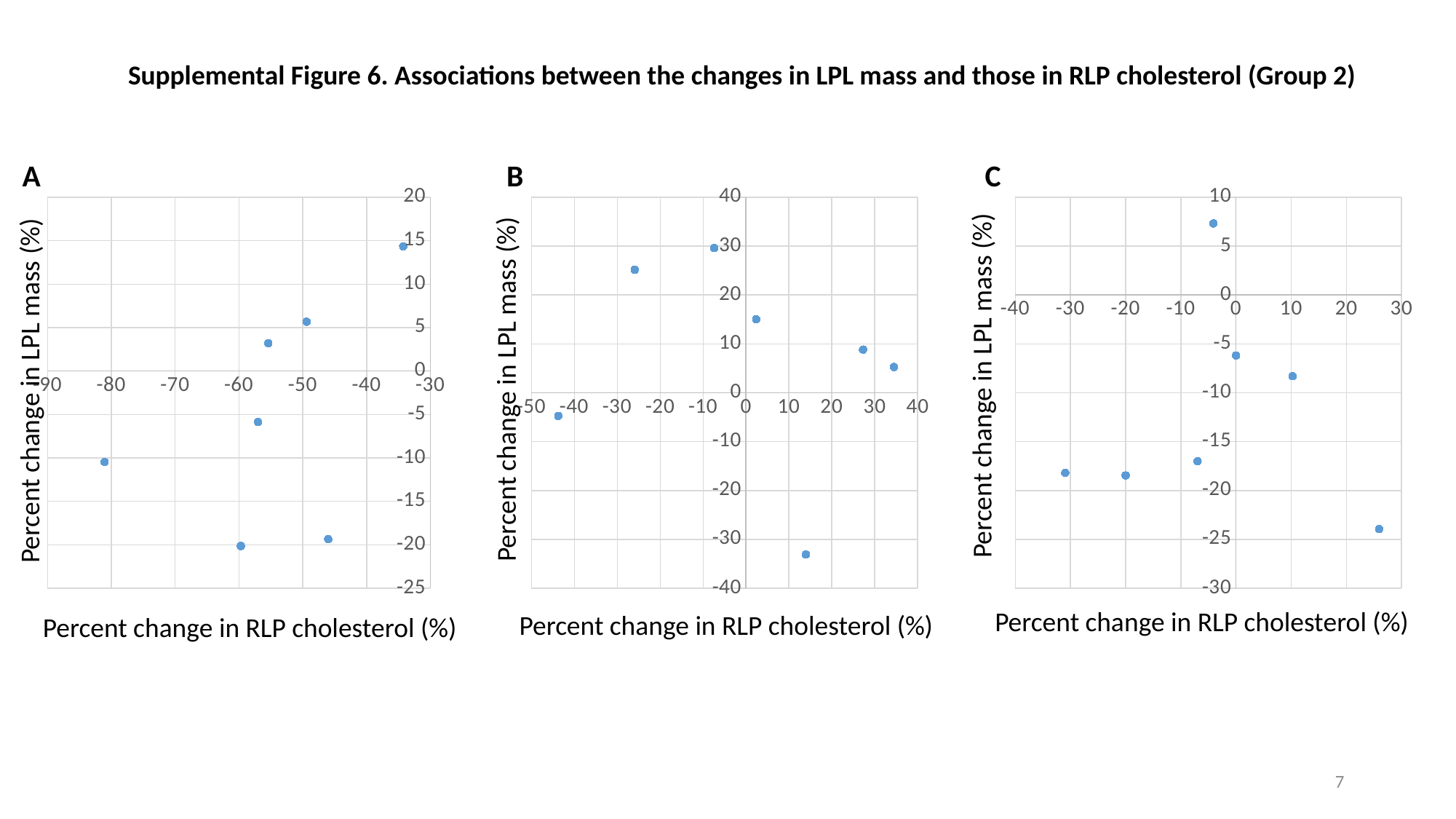

Supplemental Figure 6. Associations between the changes in LPL mass and those in RLP cholesterol (Group 2)
A
B
C
### Chart
| Category | |
|---|---|
### Chart
| Category | |
|---|---|
### Chart
| Category | |
|---|---|Percent change in LPL mass (%)
Percent change in LPL mass (%)
Percent change in LPL mass (%)
Percent change in RLP cholesterol (%)
Percent change in RLP cholesterol (%)
Percent change in RLP cholesterol (%)
7

## Slide 8
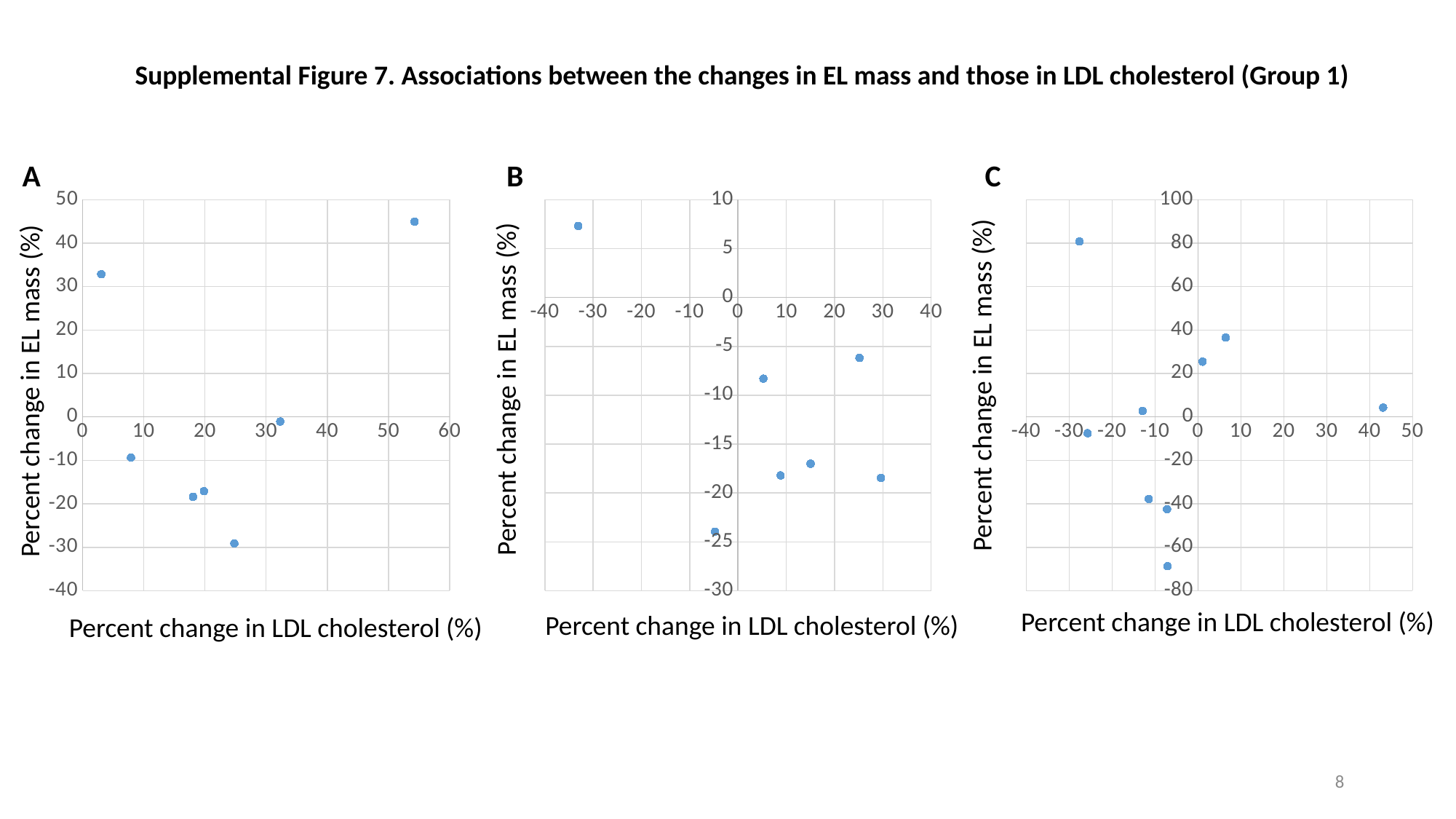

Supplemental Figure 7. Associations between the changes in EL mass and those in LDL cholesterol (Group 1)
A
B
C
### Chart
| Category | |
|---|---|
### Chart
| Category | |
|---|---|
### Chart
| Category | |
|---|---|Percent change in EL mass (%)
Percent change in EL mass (%)
Percent change in EL mass (%)
Percent change in LDL cholesterol (%)
Percent change in LDL cholesterol (%)
Percent change in LDL cholesterol (%)
8

## Slide 9
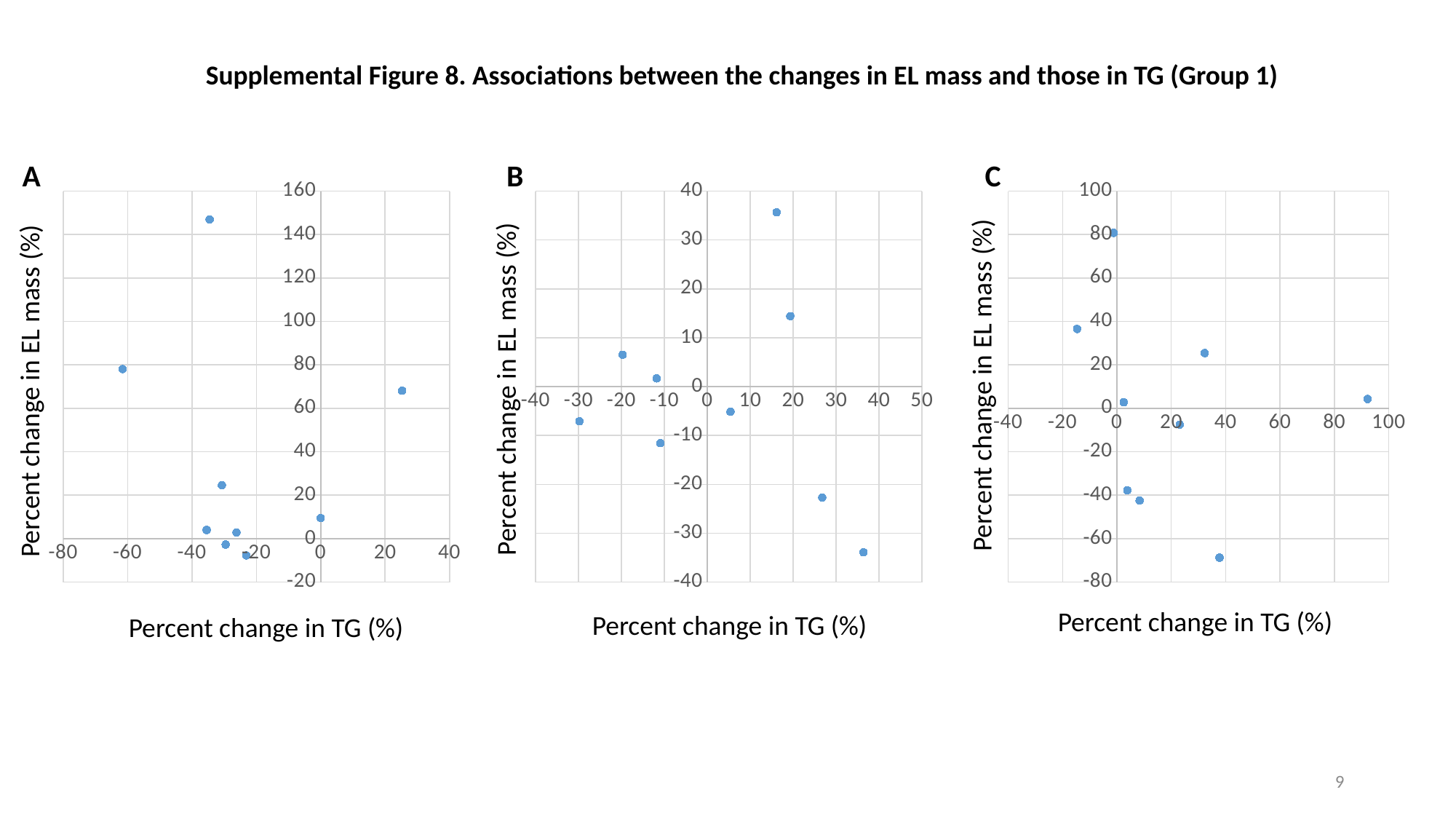

Supplemental Figure 8. Associations between the changes in EL mass and those in TG (Group 1)
A
B
C
### Chart
| Category | |
|---|---|
### Chart
| Category | |
|---|---|
### Chart
| Category | |
|---|---|Percent change in EL mass (%)
Percent change in EL mass (%)
Percent change in EL mass (%)
Percent change in TG (%)
Percent change in TG (%)
Percent change in TG (%)
9

## Slide 10
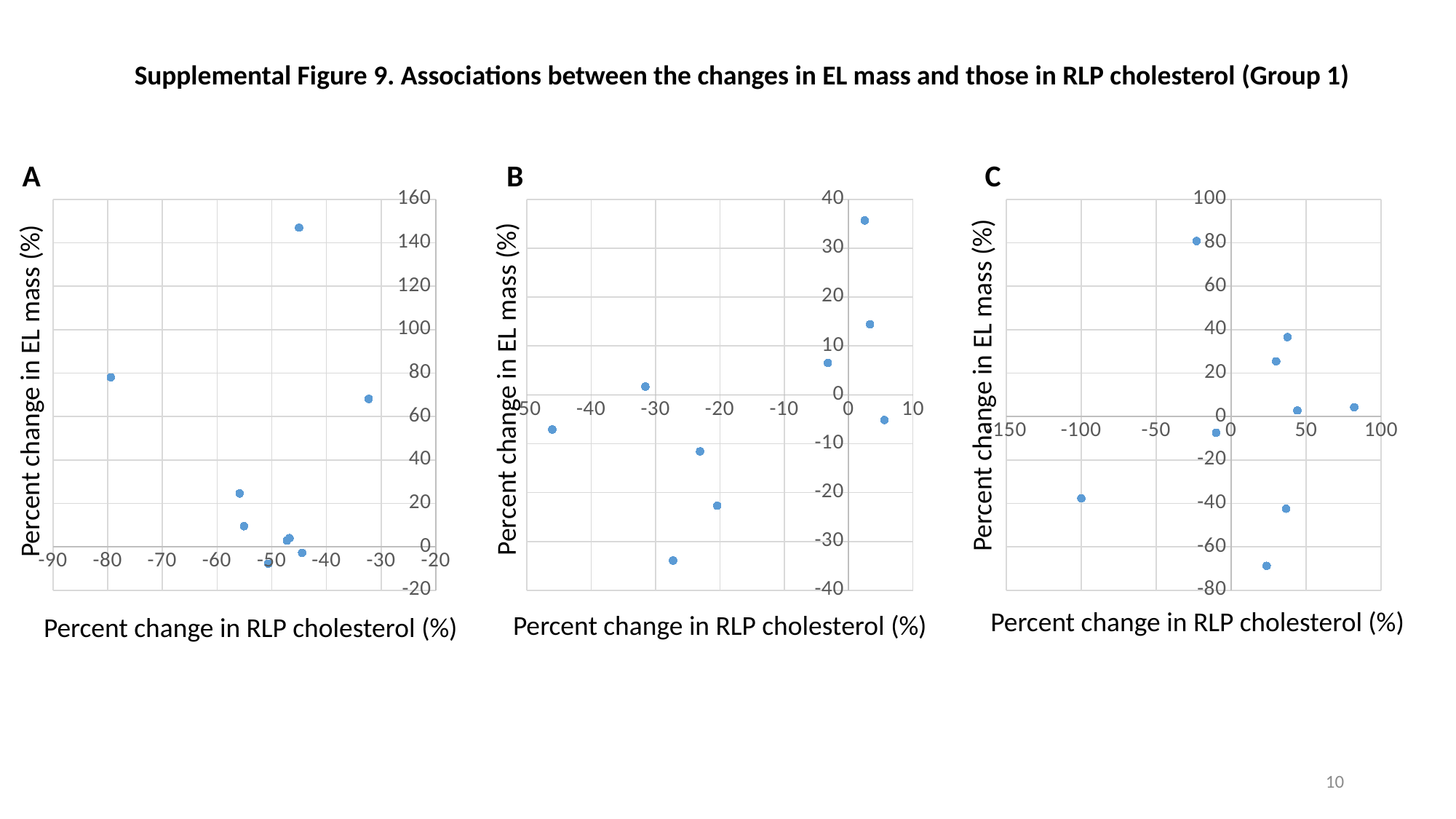

Supplemental Figure 9. Associations between the changes in EL mass and those in RLP cholesterol (Group 1)
A
B
C
### Chart
| Category | |
|---|---|
### Chart
| Category | |
|---|---|
### Chart
| Category | |
|---|---|Percent change in EL mass (%)
Percent change in EL mass (%)
Percent change in EL mass (%)
Percent change in RLP cholesterol (%)
Percent change in RLP cholesterol (%)
Percent change in RLP cholesterol (%)
10

## Slide 11
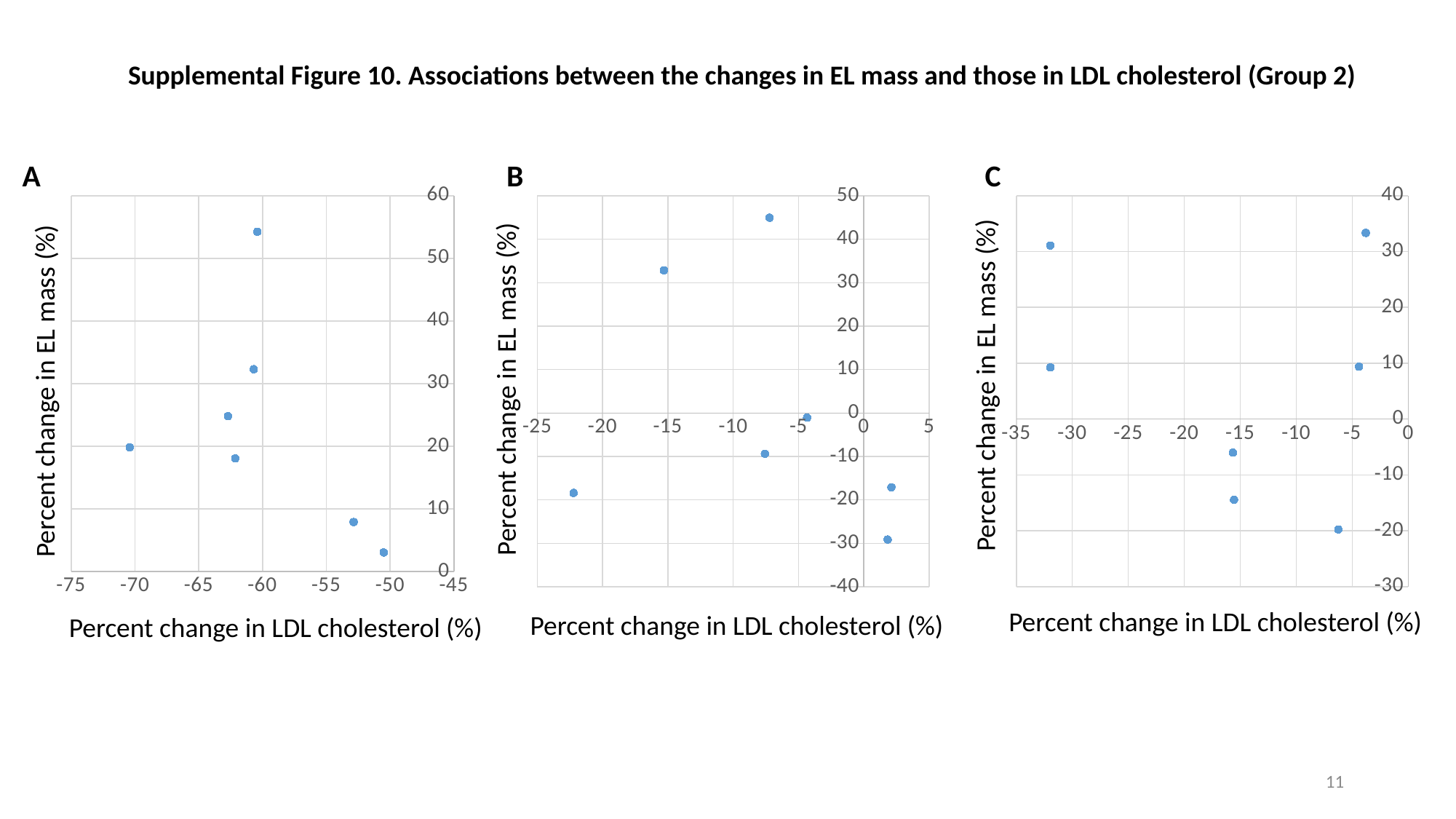

Supplemental Figure 10. Associations between the changes in EL mass and those in LDL cholesterol (Group 2)
A
B
C
### Chart
| Category | |
|---|---|
### Chart
| Category | |
|---|---|
### Chart
| Category | |
|---|---|Percent change in EL mass (%)
Percent change in EL mass (%)
Percent change in EL mass (%)
Percent change in LDL cholesterol (%)
Percent change in LDL cholesterol (%)
Percent change in LDL cholesterol (%)
11

## Slide 12
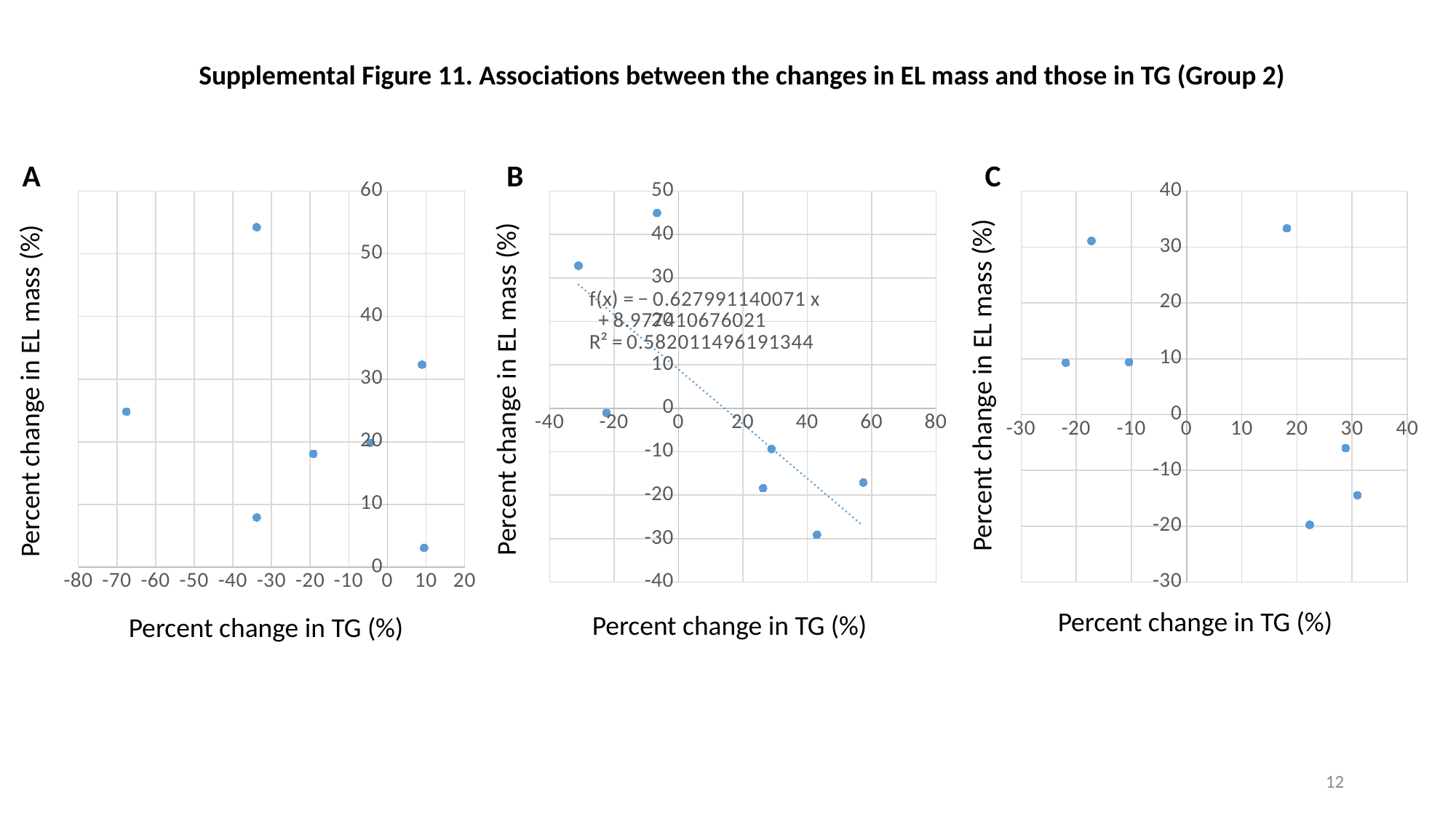

Supplemental Figure 11. Associations between the changes in EL mass and those in TG (Group 2)
A
B
C
### Chart
| Category | |
|---|---|
### Chart
| Category | |
|---|---|
### Chart
| Category | |
|---|---|Percent change in EL mass (%)
Percent change in EL mass (%)
Percent change in EL mass (%)
Percent change in TG (%)
Percent change in TG (%)
Percent change in TG (%)
12

## Slide 13
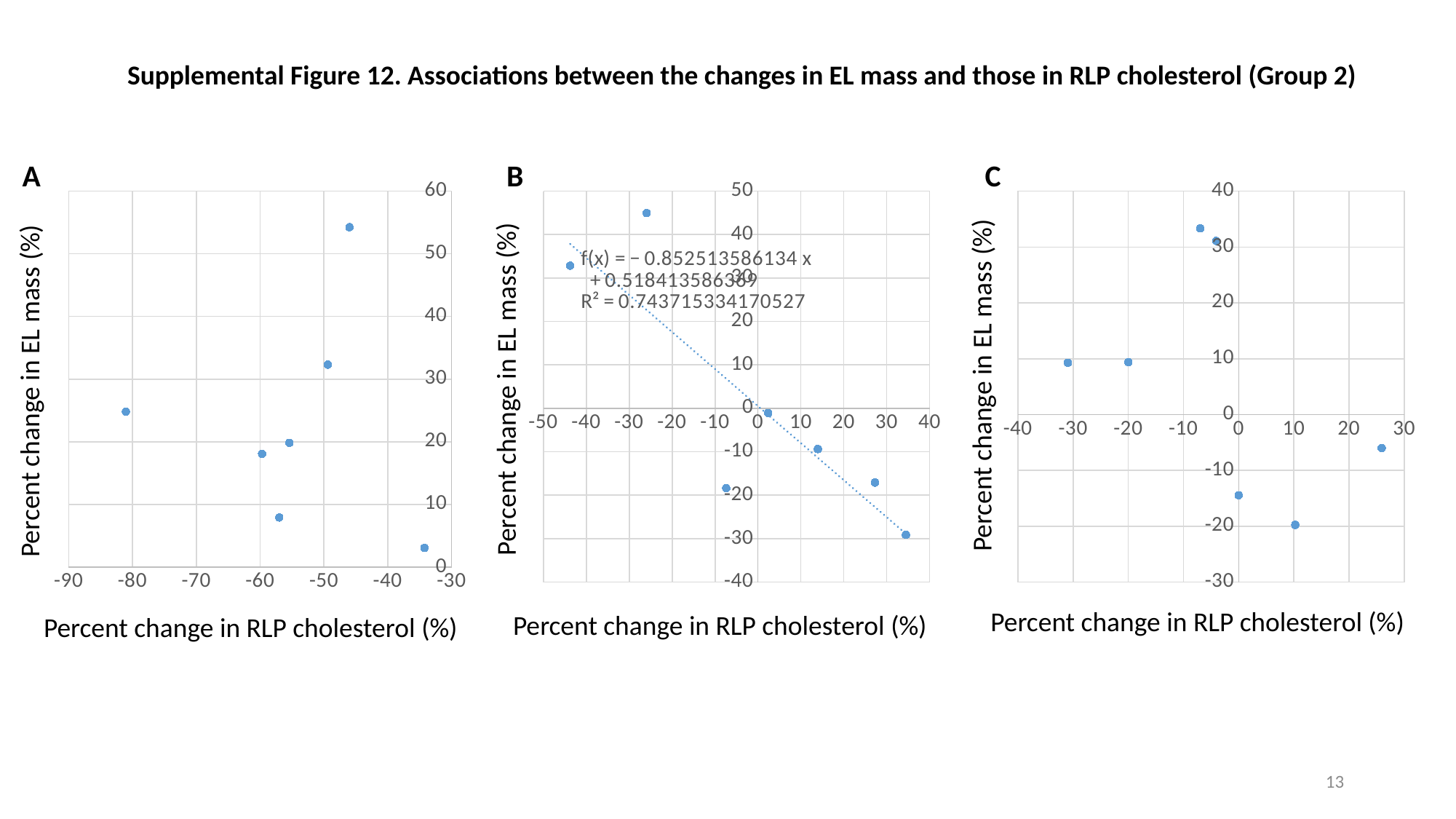

Supplemental Figure 12. Associations between the changes in EL mass and those in RLP cholesterol (Group 2)
A
B
C
### Chart
| Category | |
|---|---|
### Chart
| Category | |
|---|---|
### Chart
| Category | |
|---|---|Percent change in EL mass (%)
Percent change in EL mass (%)
Percent change in EL mass (%)
Percent change in RLP cholesterol (%)
Percent change in RLP cholesterol (%)
Percent change in RLP cholesterol (%)
13
